# Did the popularization of the Internet impact Chinese citizens’ attitude towards foreign countries? An empirical study based on two surveys

**DOI:** 10.1371/journal.pone.0291091

**Published:** 2023-09-20

**Authors:** Bowen Qin, Xiaochang Ge

**Affiliations:** School of International Studies, Renmin University of China, Beijing, China; Federal University of Technology - Parana, BRAZIL

## Abstract

Citizens’ attitudes towards foreign countries are considered an important factor in making foreign policy. This also holds in China, where public opinion is given significant weight in foreign policy-making. On the other hand, the media serves as a gateway for citizens to access the outside world, shaping their attitudes towards foreign countries. The rise of the Internet since the mid-2000s has brought about radical changes in the media landscape. The Internet, characterized by its loose control and the strong patriotic sentiment among netizens, is viewed as a breeding ground for popular nationalism. Scholars and analysts worry that the prevalence of online popular nationalism may lead to xenophobia and hostility towards Western countries portrayed as out-group others in this narrative. This study aims to investigate the impact of popular nationalism narratives flooding the Internet on citizens’ attitudes, and the differential influence of the Internet compared to traditional mass media. Furthermore, this study also examines the dynamics of citizens’ foreign attitudes and the role of media channels in shaping these attitudes. Through a quantitative analysis based on the data collected in 2010 and 2020, the study challenges concerns about xenophobic sentiments resulting from online nationalism, finding them to be unnecessary. Additionally, this study also discovers that, compared to traditional mass media, the influence of the Internet on attitude is more open to change in the international environment.

## Introduction

As an important element of public opinion, attitudes towards foreign countries act as heuristic cues when citizens evaluate their country’s foreign policy. Citizens’ attitude towards foreign countries is an unneglected element in foreign policy-making. The same is true for China, where public opinion is believed to be taken seriously during foreign policy-making. Furthermore, as the window for citizens to access the outside world, media is an important factor in shaping citizens’ attitudes towards foreign countries. Since the mid-2000s, the media environment has been experiencing radical change with the fast development of the Internet. Because of the loose control in the Internet community and the patriotic passion excited by the sense of victimhood among citizens, the Internet is viewed as a breeding ground for popular nationalism. As this phenomenon intensified, scholars, policy analysts, and observers were concerned that the prevalence of popular nationalism on the Internet is causing xenophobia and thus making people antagonistic towards the Western countries, which are the out-group others in this popular nationalism narrative. In other words, it is considered that with the spread of the Internet, the influence of the popular nationalism narrative will increase significantly and negatively impact Chinese citizens’ attitudes towards Western countries, especially Japan and the United States, that constitute others in that narrative.

Did the popular nationalism narratives that flooded the Internet significantly impact citizens’ attitudes towards foreign countries? What is the impact of the Internet on citizens’ attitudes? How does the influence of the Internet differ from the traditional mass media? What were Chinese citizens’ attitudes towards its significant opponents and partners, such as the United States, Japan, and Russia, in the 2010s? By using the data from the “Chinese View of the European Union (EU)” project, this research intends to figure out these questions. Furthermore, this research also examines the dynamics of Chinese citizens’ foreign attitudes and investigates the influence of media channels on the formation of these attitudes. Through the quantitative analysis, this research finds that concerns about xenophobic sentiments resulting from the prevalence of nationalism narratives on the Internet are unnecessary. Moreover, compared to the traditional mass media, the influence of the Internet on attitude is more responsive to the change in the bilateral relationship between China and the specific country.

In the following sections, this paper will first discuss the definition of attitude and its implication for foreign policy-making. Secondly, the changing media environment in China will be briefly introduced. Then the paper will review the debating view on the influence of changing media environment on Chinese citizens’ attitudes towards foreign countries. In the next part, quantitative research will examine those debating views. Finally, the result will be demonstrated, and future research suggestions will be given.

## Attitude and the changing media environment in China

Attitude is the feeling and effects towards a specific object, which is always considered in favour or disfavour, and the citizens’ attitude towards a foreign country can be seen as the affective feeling of individual citizens towards that country [1–3]. Both situational and dispositional elements influence the attitude formation process. In other words, attitude is a sum of information gathered from direct contact, media, and hearsay from neighbouring people, which is also mediated by an individual’s memory, background, and personality.

Acting as a hint when citizens assess their country’s foreign relations, attitude towards foreign countries has an important implication on foreign policy-making. When citizens have a positive attitude towards a given country, they intend to welcome a friendly foreign policy towards that country. On the contrary, when the attitude towards a given country tends to be negative, a more antagonistic policy is supported, especially when bilateral disputes erupt. It is easier for them to demand a more bellicose response from the government [4,5]. Thus, citizens’ attitude influences two extremes in terms of foreign policy making. On the one side, a negative attitude towards a particular country would limit the space and freedom for policy-making and make reconciliation even harder. On the other hand, a more positive attitude towards a particular country would promote more cooperative opportunities with and friendly policy towards that country.

In liberal democratic countries, elections, voting, and other political participation are significant ways for the public to influence foreign policy-making. In some authoritarian states, although the channel for political participation is limited, citizens can also express their opinion on foreign policy by demonstration, petition, and boycotting. In China, with the commercialization process of mass media and the spread of the Internet and social media, citizens have an increasing platform to receive multiple information and express their own opinion. The influence of attitude on foreign policy making is becoming significant. For instance, in 2003, Chinese citizens disrupted the Sino-Japanese relationship by initiating a petition against the two countries’ high-speed railway cooperation project [6]. Furthermore, in April 2008, the Anti-France demonstration erupted in almost all major cities in China, an important factor that made the Chinese government adopt tough measures against France on the Tibet problem [7]. Hence, public attitude is gradually becoming a factor that is hard to neglect during the foreign policy-making process in China.

Moreover, because of this, China is becoming an important target for public diplomacy, especially for Western countries. For example, many countries, including the United States, Japan, France, and even Iran and North Korea, opened their account on Weibo (the Chinese version of Twitter) and utilized it as a significant platform for conducting public diplomacy. Besides this, many countries’ embassy in Beijing also holds culture exchange activities. For instance, in 2021, the British Embassy held the Christmas Fair, the United States Embassy held an English language training program, and the Korean Embassy held a cooking class for Korean food. Furthermore, nongovernment organizations such as British Council, Japan Foundation, and Goethe-Institut funded by the home country are active in China, spreading culture, values, and language. Despite this active unfolding of public diplomacy in China, the attitudes of Chinese citizens towards those counties are the main index for judging the effect of public diplomacy [8]. Thus, the research on Chinese citizens’ foreign attitudes has important implications for the public diplomacy policy of countries that want to improve their national image in China.

Furthermore, most average people lack direct contact with the outside world and have limited means to access information. Thus, as the bridge linking individuals and the outside world, media is the major channel for citizens to access outside information and impacts the formation of citizens’ attitudes towards foreign countries. The Chinese media environment has radically changed in the last two decades. Along with the decline of traditional mass media, the Internet has become the major source of information gathering. According to the statistical report the China Internet Network Information Center released semi-annually, the number of Chinese netizens increased from 8.9 million in January 2000 to 1.01 billion in June 2021 [9,10]. However, because of the different nature between the Internet and mass media, it is widely concerned that the spread of the Internet would impact the attitude towards foreign countries. The following section will discuss the nature of the Internet and its impact on public attitude.

## The spread of the internet and the prevalence of popular nationalism

On the international stage, nation-states are the main actors. And culture serves as a significant indicator that distinguishes different actors. According to Sauquet and Vielajus, cultures vary in their perceptions of tradition and modernity, nature, time, work, and equality [11]. Hofstede proposes five dimensions to measure cultural differences: power distance, uncertainty avoidance, individualism/collectivism, masculinity/femininity, and long-term orientation [12]. These cultural disparities give rise to distinctions among groups, often formed by nation-states [13]. Moreover, the varying nature of relationships, ranging from enemies to hostility to friendship, further magnifies the differences, resulting in the formation of in-groups and out-groups. In-group countries are always favoured, while out-group countries are subjected to bias [14,15]. At the individual level, this trend of in-group favouritism and out-group bias generated nationalist sentiment and engendered a preference for in-group countries while harbouring animosity towards out-group countries [16,17]. This is particularly evident in China, where the collective cultural context leads individuals to prioritize affiliation, thereby contributing to the prevalence of popular nationalism and group differentiation [18,19]. On the other hand, the information accessibility, individualized, and emotional feature of the Internet makes it the breeding ground for popular nationalism, and it is thus concerned that the spread of the Internet would harm Chinese citizens’ attitude towards foreign countries. Especially the Western countries that invaded China during the “Century of Humiliation,” such as the United States and Japan, are considered the out-group countries in the Chinese historical narratives.

Firstly, the spread of the Internet widened the transmission and access channel of information. Every individual can post and receive information from the Internet. This trend broke the monopoly of traditional mass media on information and impacted the news management system of the government. Secondly, social media linked the public separated geographically and enabled a relatively free environment for opinion expression and information sharing [20]. The spread and development of Internet media, characterized as free, open, and plural, challenged the opinion environment dominated by traditional media, and traditional media is not the only means for the masses to obtain outside information anymore. The citizens’ trust in the information resource changed from dependent on authority to reliability, and thus, the traditional media is experiencing a “trust crisis” [21]. At the same time, Shi’s research reveals that when the information transmitted by the traditional media conflicted with the citizens’ opinion, they would turn to the Internet to obtain more information [22]. Thus, the more frequently individuals access the Internet, the more resistant he or she would be to the information transmitted by the traditional media. Furthermore, the spread of the Internet facilitated the expression of emotions such as anger, making nationalism an important factor influencing Internet opinion and mobilization [23,24]. Moreover, the Internet has become a significant platform for companies to advertise their products or services. To garner attention, these companies often employ nationalism as a marketing strategy, associating their offerings with a sense of national pride. This phenomenon has led to the commercialization of nationalism, thereby amplifying the influence of nationalism [25,26]. In summary, the Internet has created a fertile ground for the emergence and proliferation of nationalism [27].

Those Internet features are the breeding ground for Chinese popular nationalism, a phenomenon that has emerged since the 1980s. This rise of nationalism is stimulated by the increasing freedom of expression brought by market reform and the campaign to implement patriotic education [28,29]. Even though the content of Chinese popular nationalism is complicated and under constant debate in academia, the discourse is mainly focusing on three elements: (1) a hundred years of humiliation, (2) the strong sense of victimhood caused by the history of humiliation, (3) the wish of a prosperous country with a powerful army [30–33]. Because of the vivid contrast between the glorious past of hegemony in the tribute system and the disgraceful experience of humiliation by the Western countries, there is a strong aspiration in the Chinese nationalist discourse to get back to the top of the hierarchy and wash away the humiliating past. Thus, as Whiting argued, the Chinese nationalist discourse is constituted by three faces, which are affirmative (to positive in-group attribute), assertive (to negative out-group), and aggressive (to identified out-group enemies) [34]. In other words, there are strong in and out-group distinctions in the Chinese nationalist discourse, and it is concerned that the rise of Chinese nationalism would bring xenophobia, causing a negative attitude towards out-group foreign countries among Chinese citizens. Specifically, the spread of the Internet might worsen Chinese citizens’ attitudes towards countries that invaded China in the past or are now confronting China.

Some scholars thus believe that the Internet facilitates the influence of Chinese popular nationalism, making Chinese citizens increasingly antagonize foreign out-group countries [29,35–42]. For example, Reilly argues that the popular nationalism transmitted on the Internet is extreme and hard to control by the Chinese government [29]. Gries holds almost the same view as Reilly and points out that the Internet nationalism targeted at Japan is the second wave of Chinese nationalism after the anti-America nationalism mainly based on books and magazines [36]. Furthermore, Rozman reveals that after the late 1990s, because of the prevalence of nationalist sentiment facilitated by the Internet, the Chinese government found it hard to reshape the anti-Japanese element in the nationalist discourse [35]. Their research analysing opinions expressed on Chinese Internet platforms like Weibo, Weiss, Wu, and Sullivan and Wang found that Chinese netizens exhibit a hawkish stance [37,41,42]. They demonstrate greater confidence in China, support aggressive foreign policies, and endorse military development and investment. Additionally, there are researches investigating how netizens are mobilized by popular nationalism on the Internet during the public incident [43,44].

On the other hand, another group of scholars argues that there is no direct link between the spread of the Internet and the rise of Chinese popular nationalism, and the nationalist discourse on the Internet demonstrates a trend of heterogeneity and mainly targets the weakness of Chinese government but not the foreign enemy. Cairns and Carlson investigated the opinion and public discussion on Weibo during the 2012 Diaoyu/Senkaku Island dispute. They found that the expression and emotion on Weibo are not mainly occupied by nationalist sentiment [45]. The moderate voice also had its space on Weibo, and the harshest nationalist expression was not targeted in Japan but the Chinese government, which was corrupted and weak. Furthermore, by analysing the Internet behaviour of Chinese urban elites, Guo argues that they exhibit characteristics of critical nationalists [46]. These individuals are calm, intellectual, and display a lower sense of xenophobia. Zhang, Liu, and Wen also challenged the claim that the Internet is flooded with nationalism and argued that most discussions on the Internet focus on criticizing the Chinese government [47]. Moreover, even the nationalism discourse on the Internet is complicated and pluralized, and different nationalists have a different understanding of who is the enemy of contemporary China. Wang argues that there are different understandings of Mao’s famous motto, “The backward will be beaten,” on the Internet between leftists and liberals [48]. Duan adds that nationalism is not the main topic discussed on the Internet, with the top search record being domestic affairs [49]. Furthermore, Johnston argues that no substantial evidence supports that nationalism in China is rising [50].

In sum, it is clear that there are two separate views of the spread of the Internet. One group of scholars argues that Chinese nationalism is further intensified by the spreading Internet, leading to the deterioration of attitudes towards out-group foreign countries. In other words, if citizens access the Internet more frequently, he or she would have a more negative attitude towards the out-group foreign countries and a more positive attitude towards in-group foreign countries.

**Hypothesis 1:** The more frequent access to the Internet, the more negative attitude towards out-group countries and positive attitudes towards in-group countries.

Furthermore, it is believed that the discourse of popular nationalism has been stable and constant for a relatively long period. In other words, they have a clear and stable version of enemies and friends, and this view is hard to change without a massive transformation in the international environment, such as war or reconciliation. Thus, if an individual access the Internet more frequently, he or she would be insensitive to the non-substantial change in the international environment.

**Hypothesis 2:** The more frequent access to the Internet, the greater the level of consistency in attitudes towards foreign countries, particularly when there are no substantial changes in the international environment.

However, as mentioned above, another group of scholars believes that the spread of the Internet only has limited influence on Chinese popular nationalism.

**Hypothesis 3:** There is no positive relationship between the frequency of Internet access and negative attitude towards out-group countries and positive attitudes towards in-group countries.
**Hypothesis 4:** There is no negative relationship between the frequency of Internet access and attitude change.

Based on those hypotheses, this research intends to join the debate on the influence of the Internet on the rise of Chinese popular nationalism based on two survey data of “Chinese view on European Union (EU)” conducted in 2010 and 2020, and taking the Chinese citizens’ attitude towards Japan, the United States, and Russia as the dependent variable for analysing the influence of the Internet.

These three countries hold great importance on the global stage, and their relationships with China carry significant implications. China’s relationship with these countries represents three distinct types of bilateral relationships, which can be categorized into two different social relationships, making them ideal subjects for comparison and analysis. The United States (US) is currently in a comprehensive confrontation with China across various domains, including ideology, values, geopolitics, technology, and military development, thereby representing a significant out-group country in the Chinese perception. Japan, the most important ally of the US in the Asia-Pacific region, has a history of committing grievous war crimes against China and is engaged in maritime territorial disputes and strategic competition with China in East Asia, making it another notable out-group country. On the other hand, Russia, as a major power with nuclear capabilities and abundant natural resources, serves as a strategic rival to the United States. Additionally, being China’s largest neighboring country, Russia’s historical association with Communism during the Soviet Union era fosters a sense of affinity among Chinese citizens. Given the intensifying Sino-US confrontation, Russia is increasingly perceived as an important strategic partner for China, making it an in-group country in the Chinese view. By categorizing Japan and the United States as out-group countries and Russia as in-group countries, this research aims to investigate the influence of the Internet on nationalist sentiment in the following section.

## Material and methods

The data of this study is from two public surveys of the Project of Chinese View of the European Union. The two surveys were completed in July, August 2010 and November 2020, respectively, and conducted in six typical Chinese cities: Beijing, Shanghai, Guangzhou, Chengdu, Xi’an, and Nanning. The respondents were all urban residents aged 18 to 74 with local household registration. A multi-stage probability proportional to size sampling method was adopted to select the sample. Specifically, the sampling process for the sample was as follows: Probability Proportional to Size (PPS) sampling method was used to identify each district (three to four districts depending on their share of the city’s population), street (three to four per district) and community (two per street) drawn from each city, which resulted in 24 (3x4x2 or 4x3x2) communities per city; at the community level, the group applied equally spaced sampling was used to select households (sample size of no more than 21 per community, but calculated with a sample size of 30 in the systematic sampling process to prevent blanking), and the household member whose birthday was closest to June 1 was selected face to face to answer the questionnaire. The data all passed ethical review. The specific sample distribution is detailed in Appendix A. The 2010 questionnaire consisted of 130 questions, including the respondents’ attitudes, knowledge, interests, and perceptions of the EU and their attitudes and perceptions of some major countries such as the United States, Japan, and Russia. It also investigated the respondents’ media use, sociocultural values, and personal information. A total of 3019 valid questionnaires were collected. The 2020 questionnaire, based on the 2010 one, investigated the respondents’ attitudes and perceptions of more countries such as Switzerland, India, and South Korea. Besides, it added questions concerning the participation possibility of different countries in the Belt and Road Initiative and public evaluation of the fight against COVID-19. A total of 3000 valid questionnaires were collected.

First, 32 variables were selected from each of the two surveys according to their relevance to this study. These include respondents’ attitudes towards the US, Japan, and Russia, media use, background, etc. Two new databases by year were also created for the comparative analysis of this study. The software used for the econometric analysis in this study was SPSS 26.0. The following two sections will conduct a comparative study based on two dimensions: firstly, the country dimension, in which this study will systematically compare the attitudes of the Chinese public towards three different countries: the United States, Japan, and Russia; secondly, the channel dimension, as mentioned earlier, in the past two decades, the media environment experienced huge change, the Internet has increasingly become the main channel for people to obtain information about the outside world in recent years. This research also intends to investigate the influence of different media channels on the Chinese public attitudes towards foreign countries. Because of this, we will compare the similarities and differences between the Chinese public’s responses to the 2010 and 2020 surveys.

Combining the theoretical hypothesis and the sample, we focus on the following variables:

### Explained variable

#### Foreign attitudes

Referring to the previous definition of attitudes, we asked respondents whether they had favourable or unfavourable impressions of the US, Japan, and Russia in the questionnaire. The Chinese word “impression” has a stronger affective dimension than English. The indicator has two dimensions, with 0 representing “unfavourable impression” and 1 representing “favourable impression.”

### Explanatory variables

#### Media use

Different media platforms have distinct characteristics, preferences, and implications for information recipients. Respondents were asked to answer separately how often they used each of the four media, TV, newspaper, radio, and Internet, in the past week, using a scale ranging from 0 to 7 days. To differentiate between the Internet and traditional mass media and to account for the variability in the proportion of mass media usage, we referred to the frequency of television usage named as Mass media uses. Meanwhile, the frequency of Internet use was named separately as Internet media uses.

#### Social identity theory

Do differences in identification with different countries lead to different attitudes towards them? Is there a distinction between in-group and out-group countries? To address these questions, we introduce social identity theory (SIT) to explain some variables. Social identity theory suggests that the quest for self-esteem drives individuals to join groups and seek positive self-identity [51,52]. As an important attribution of an individual’s group identity, the state is the main unit through which individuals engage in in-group positivity and intergroup competition [53]. Pursuing a positive group identity exacerbates interstate rivalries and induces international conflict. Regarding the definition, social identity theory operates through the following variables:

#### National identity

Research on social identity theory suggests that the more positive the perception of and affection for one’s own country, the more likely one is to have negative attitudes towards other countries [54]. Therefore, we added the national identity variable to the model and asked respondents to evaluate the statement “I am proud to be Chinese” with the options of Strongly disagree (1), Disagree (2), Agree (3), Strongly agree (4).

#### Political and social satisfaction

We asked the respondents to rate the government from the aspects of “economy,” “employment,” “environment,” and “social welfare.” The options were Not satisfied at all (1), Not so satisfied (2), Fairly satisfied (3), and Very satisfied (4). Afterward, we averaged these four variables to obtain a new variable named Political and social satisfaction.

#### Trust other nations

Some studies have found that trust in other countries contributes to positive intergroup attitudes [55]. Therefore, the model was examined by adding the variable of trust in other countries. Respondents were asked to rate the statement “Generally speaking, China can trust other nations.” with the options of Strongly disagree (1), Disagree (2), Agree (3), and Strongly agree (4).

In addition, from the perspectives of international relations theory, psychological theory, and other related theories, an individual’s socio-demographic factors affect their perceptions, impressions, and judgments about a country (or region) [56]. Previous studies on the factors influencing citizens’ image of a country also examine such variables. Chen et al. conducted a study in 2011 that also used some data from this study and confirmed that the international trust of Chinese urban residents varies with gender, income, and education [57]. Han finds that variables such as gender, personal income, education, and region affect the Chinese citizens’ favourable image towards Japan based on data from the Chicago Global Commission in 2006 and 2008 [58]. A study by Dong proves that annual income level and whether one has been to an EU country may influence Chinese scholars’ attitudes towards the EU [59].

Moreover, two surveys conducted by the Institute of European Studies of the Chinese Academy of Social Sciences in 2007 and 2008 also concluded that differences in social class, occupation, age, and gender could cause differences in groups’ perceptions of the EU [60,61]. In addition, Johnston and Stockmann studied Chinese citizens’ image of the United States [62]. They found that well-educated Chinese citizens with overseas experience are more likely to express favourable feelings towards the United States. Based on the above findings and considering the covariance between some of the variables and media use, the following five variables are selected as control variables to be recorded and added to our model: **Gender** (0 = female, 1 = male), **Education level**, **the Communist Party membership** (0 = not Party member, 1 = Party member), **Social status scale** (where would you put yourself on the social status scale? 1 to 10, 1 = bottom, 10 = top), **Overseas experience** (0 = no, 1 = yes), and **Experience of contact with foreigners** (0 = no, 1 = yes).

## Result

### Descriptive analysis

**Fig 1:**
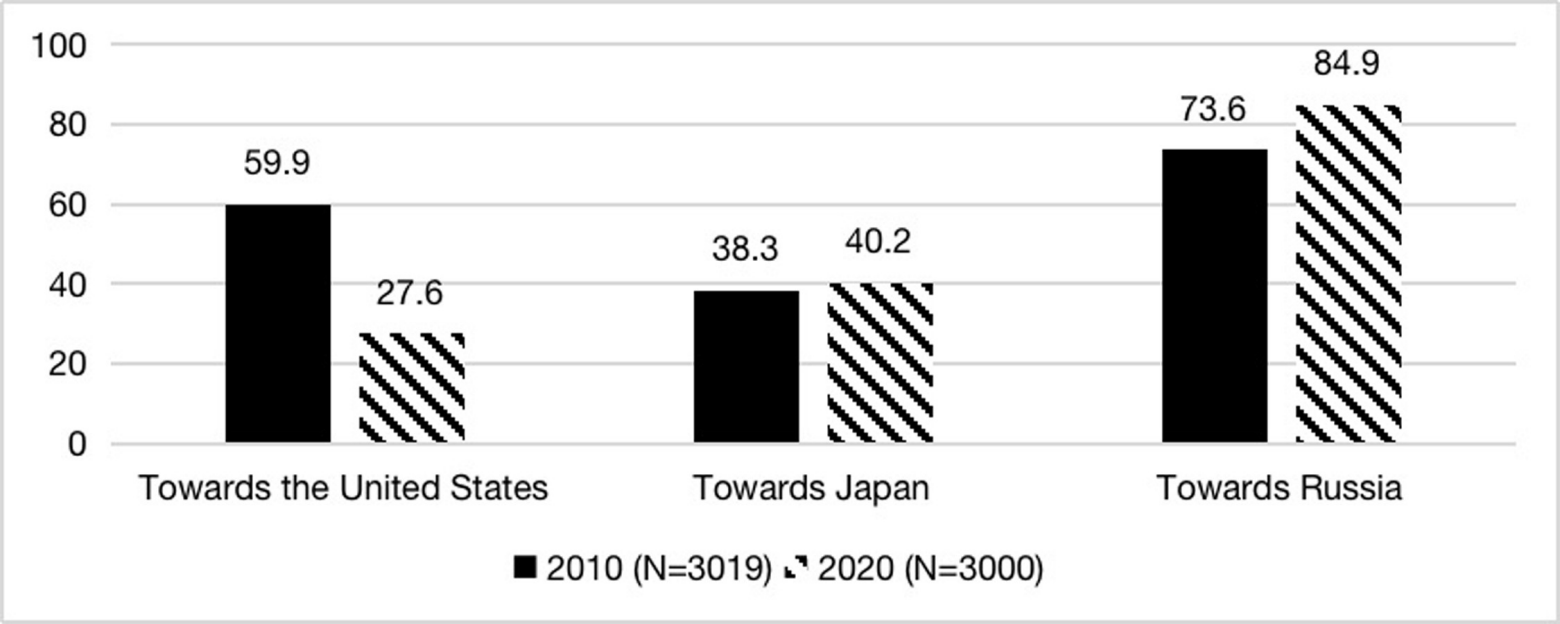
Favourable impression towards the United States, Japan, and Russia among Chinese Citizens (%).

First, we performed a descriptive analysis of Chinese public attitudes towards other countries, shown in Fig 1. In 2010, most respondents had a favourable impression of the United States and Russia. Nearly three-quarters of respondents had a positive attitude towards Russia. Around 60% of respondents had a positive attitude towards the US; Japan was relatively low, with only 38.3% expressing a positive attitude. Ten years later, in 2020, the sample’s favourability towards Russia increased, reaching 84.9%. Favourability towards Japan has not changed much compared to ten years ago, with an increase of only 1.9 percent. Since we did not track the same respondents for the decade, we cannot capture the dynamic change of the favourable attitudes towards Japan among the same people. Nevertheless, it was predictable that with the outbreak of territorial disputes and conflicts over historical issues between China and Japan since 2012, Chinese citizens’ favourable attitudes towards Japan in 2020, to some degree, resulted from the thawing of Sino-Japanese relations. This result was confirmed by the Public Opinion Survey on China-Japan Relations jointly conducted by the China Foreign Affairs Bureau and the Japan Speech NPO. According to this survey, in 2010, 38.3% of Chinese participants said they had a favourable opinion of Japan, and in 2014 this plummeted to 5.3%. While in 2019, the percentage rose back to 45.9%, which was similar to our data [63]. As for the Chinese public favourable attitudes towards the United States, with the negative impact of the Trump administration’s policies towards China, the percentage of favourable attitudes dropped significantly from 59.9% in 2010 to 27.6% in 2020, making the United States the least favourable country for Chinese citizens. Similar findings were found in the Global Times’ Chinese People’s View of the World survey. In the question “Which is your favourite country (choices including China),” the percentage of respondents choosing the United States as their favourite country dropped from 7.5 percent in 2010 to less than 2 percent in 2020 less than 2% in 2020 [64].

**Fig 2:**
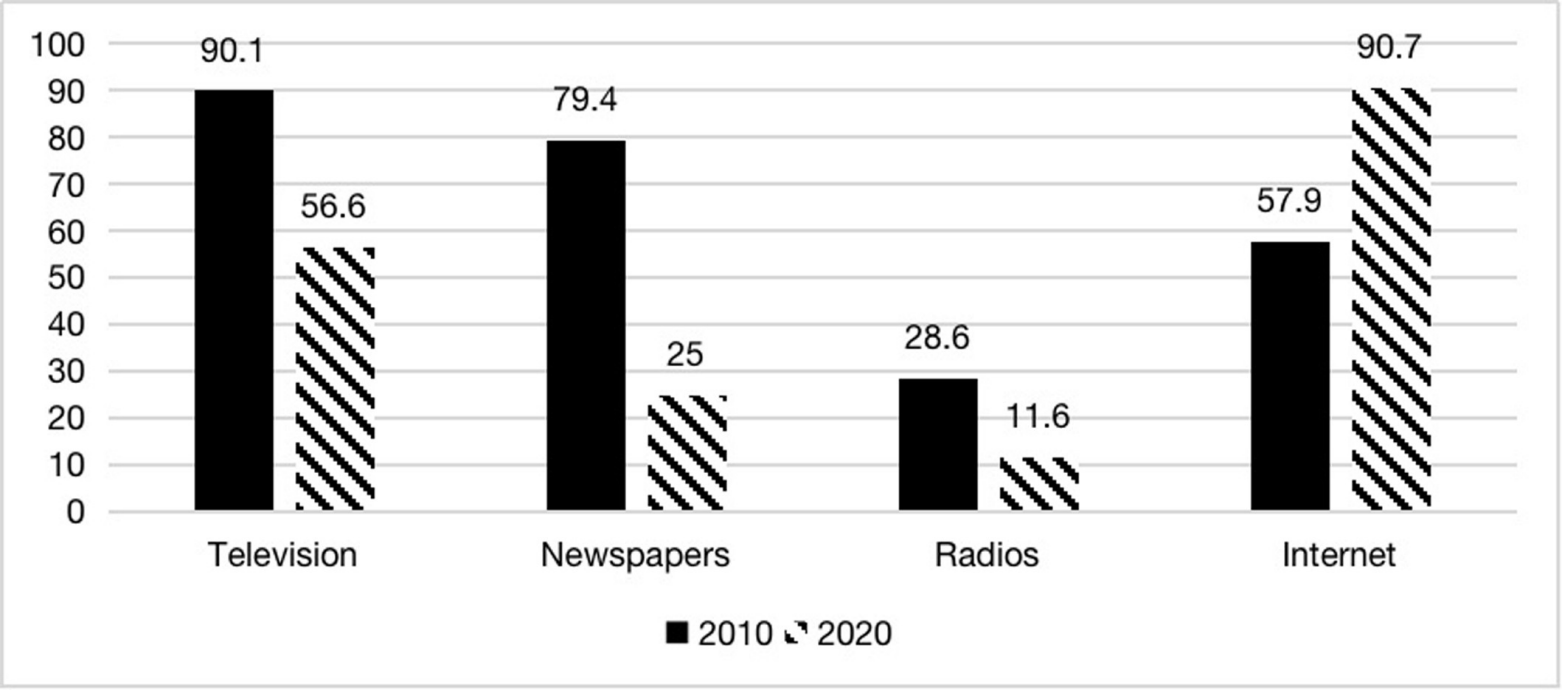
Number of media users in the past week (%).

**Fig 3:**
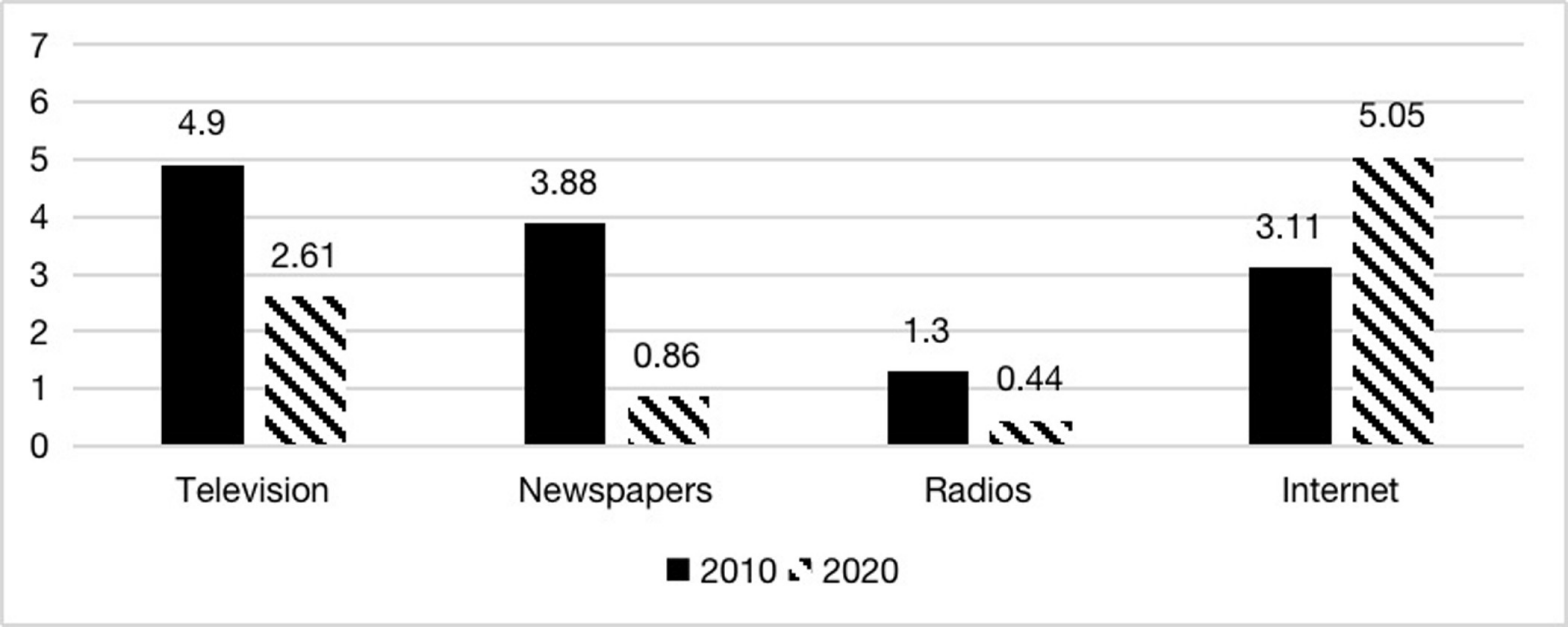
Using the frequency of different media in the past week (average days).

Internet media has grown leaps and bounds over the past decade, while traditional media has declined. Is this trend reflected in the sample? The results are shown in Figs 2 and 3. Television was the most widely used traditional media in 2010. Regarding the number of users, more than 90% of the respondents indicated that they watched television in the past week, 80% for newspapers which second only to television, and 57.9% for the Internet. The radio, a more traditional form of media, was used by less than 30% of the respondents in the past week. With the rapid growth of the Internet and new media from 2010 to 2020, the number of users using these four media in 2020 changed significantly. More than 90% of the respondents used the Internet in 2020, making it the most used media. While the number of respondents using television dropped to 56.6%. The most notable change was in newspapers, which plummeted from nearly 80% a decade ago to 25% in 2020, dropping 54.6%. Similarly, radio use declined by 17%, with only about one-tenth of the respondents still using radio for information in 2020.

The weekly using frequency of use of these four media was consistent with the above findings: television and newspapers were the most important channels for respondents to acquire information in 2010, followed by the Internet; while in 2020, the use frequency of the Internet far exceeded that of other media. According to the result, the respondents read newspapers and used the radio for less than one day a week on average, which means that a significant proportion of respondents who had access to newspapers and radio in the past week may not use these two media as a regular tool for acquiring information. In addition, one question in the questionnaire asked the respondents about the most important information sources for the European Union. In 2010, the top five votes were for television, newspapers, the Internet, radio, and books. In 2020, the Internet jumped to the top, with television and books in second and third place. Although the questionnaire did not include similar questions about other countries, we cannot make a cross-sectional comparison here. However, it was still possible to draw similar conclusions as in Figs 2 and 3. Firstly, the media channels used by the population for external information have become more homogeneous than they were ten years ago (the average number of types of media used per week has fallen from 2.65 to 2.17). That is, there is a greater concentration on accessing information via the Internet. Secondly, traditional media have become less important as a medium for Chinese people to access information. While still important, television has declined significantly from its share a decade ago. Newspapers, one of the most important media for Chinese people to learn about the world ten years ago, are now irrelevant, and very few people still use the radio. Finally, the Internet and new media use have increased dramatically this decade. In particular, new media platforms such as WeChat and Weibo are becoming the main channels for Chinese people to learn about the world.

### Regression model analysis

Do exposure to different media types, which are the Internet and traditional media, impact people’s attitudes differently? To be specific, as Internet exposure increases, are people more likely to receive nationalistic influences that lead to more hostile attitudes towards out-group countries and more friendly attitudes towards in-group countries? Does the influence of different media channels change over time? Based on descriptive statistics, we attempted to apply logistic regression analysis to explain these questions. As the dependent variable selected for the model is dichotomous, we used binomial logistic regression analysis to build the statistical model. With the inclusion of separate control variables and the addition of the core explanatory variable of media usage, the specific regression results are shown in Tables 1 and 2, with each column representing a separate regression equation. The dependent variables in Models 1 and 2 are attitudes towards the US, Models 3 and 4 are attitudes towards Japan, and Models 5 and 6 are attitudes towards Russia.

**Table 1:** Differential influences of different media channels on Chinese citizens’ attitudes in 2010.

	Towards the United States		Towards Japan		Towards Russia	
	(1)	(2)	(3)	(4)	(5)	(6)
Gender	-0.130	-0.125	-0.145*	-0.136	-0.067	-0.038
	(0.086)	(0.089)	(0.085)	(0.087)	(0.104)	(0.107)
Education (in years)	0.030**	0.024	-0.031**	-0.001	-0.002	0.019
	(0.014)	(0.017)	(0.014)	(0.017)	(0.018)	(0.020)
Party membership	-0.211**	-0.220**	0.092	0.078	0.079	0.113
	(0.102)	(0.105)	(0.101)	(0.104)	(0.124)	(0.129)
Social status scale	0.114***	0.095***	0.072***	0.062**	-0.064*	-0.064*
	(0.028)	(0.029)	(0.028)	(0.029)	(0.034)	(0.035)
National identity	-0.221***	-0.206***	-0.206***	-0.227***	0.321***	0.337***
	(0.071)	(0.073)	(0.071)	(0.073)	(0.079)	(0.081)
Trust other nations	0.234***	0.272***	0.359***	0.358***	0.174**	0.180**
	(0.069)	(0.072)	(0.070)	(0.072)	(0.083)	(0.086)
Political and social satisfaction	0.257***	0.236**	0.528***	0.484***	0.460***	0.449***
	(0.089)	(0.093)	(0.091)	(0.094)	(0.107)	(0.111)
Overseas Experience	-0.030	-0.079	0.113	0.073	-0.176	-0.148
	(0.122)	(0.125)	(0.119)	(0.121)	(0.140)	(0.143)
Experience of contacting with foreigners	0.352***	0.342***	0.124	0.207**	-0.057	-0.003
	(0.097)	(0.101)	(0.095)	(0.099)	(0.114)	(0.119)
Mass media uses		0.049***		0.054***		0.004
		(0.019)		(0.019)		(0.022)
Internet media uses		0.038**		-0.056***		-0.058***
		(0.017)		(0.017)		(0.021)
Constant	-0.717*	-1.001**	-1.740***	-2.003***	-0.969**	-1.143**
	(0.374)	(0.391)	(0.375)	(0.391)	(0.432)	(0.453)
						
Number of samples	2,491	2,376	2,468	2,354	2,418	2,302
Pseudo R-squared	0.0245	0.0296	0.0321	0.0394	0.0303	0.0355

Note: Standard errors in parentheses

*** p<0.01

** p<0.05

* p<0.1.

Source: Made by the authors.

**Table 2:** Differential influences of different media channels on Chinese citizens’ attitudes in 2020.

	Towards the United States		Towards Japan		Towards Russia	
	(1)	(2)	(3)	(4)	(5)	(6)
Gender	0.031	-0.019	-0.130	-0.154*	-0.127	-0.106
	(0.089)	(0.100)	(0.080)	(0.089)	(0.111)	(0.126)
Education (in years)	0.034**	0.030*	0.031**	0.018	-0.013	-0.005
	(0.016)	(0.018)	(0.014)	(0.016)	(0.020)	(0.022)
Party membership	0.224	0.311**	-0.158	-0.118	0.458**	0.444**
	(0.137)	(0.147)	(0.128)	(0.137)	(0.202)	(0.222)
Social status scale	0.089***	0.054	0.068**	0.092***	-0.040	-0.012
	(0.030)	(0.035)	(0.027)	(0.031)	(0.037)	(0.043)
National identity	-0.884***	-0.850***	-0.537***	-0.620***	0.645***	0.757***
	(0.081)	(0.096)	(0.076)	(0.091)	(0.089)	(0.108)
Trust other nations	0.415***	0.474***	0.346***	0.394***	0.104	0.203**
	(0.064)	(0.074)	(0.057)	(0.065)	(0.079)	(0.091)
Political and social satisfaction	-0.042	-0.036	-0.067	-0.083	0.401***	0.352***
	(0.092)	(0.105)	(0.082)	(0.093)	(0.111)	(0.128)
Overseas Experience	-0.080	0.002	0.079	0.111	-0.313**	-0.385***
	(0.112)	(0.121)	(0.101)	(0.109)	(0.133)	(0.146)
Experience of contacting with foreigners	0.311***	0.329***	0.218**	0.212**	0.082	0.018
	(0.098)	(0.107)	(0.089)	(0.095)	(0.125)	(0.137)
Mass media uses		-0.045*		-0.036*		-0.025
		(0.023)		(0.020)		(0.028)
Internet media uses		-0.116***		0.069***		0.024
		(0.027)		(0.025)		(0.034)
Constant	0.227	0.834	0.101	0.159	-1.495***	-2.270***
	(0.433)	(0.515)	(0.401)	(0.476)	(0.511)	(0.613)
						
Number of samples	2,828	2,313	2,823	2,311	2,801	2,299
Pseudo R-squared	0.0664	0.0755	0.0345	0.0431	0.0459	0.0550

Note: Standard errors in parentheses

*** p<0.01

** p<0.05

* p<0.1.

Source: Made by the authors.

The model results, which first introduce control variables and social identity theory explanatory variables, show that the factors influencing Chinese people’s favourable attitudes towards the US and Japan are relatively similar in both 2010 and 2020, while the factors influencing favourable attitudes towards Russia are the opposite. Specifically, in 2010, respondents who perceived themselves as having a higher social status were more likely to have positive attitudes towards the US and Japan but more negative attitudes towards Russia. Respondents with a higher identification with their home country are more negative in their attitudes towards the US and Japan but more positive in their attitudes towards Russia. The experience of going abroad also increases respondents’ positive feelings towards the US and Japan, but not significantly towards Russia. In 2020, the impact of national identity is more similar to that in 2010. Subjective social status still positively affects attitudes towards the US and Japan, while the effect on attitudes towards Russia is no longer significant. Experience abroad no longer affects attitudes towards the US and Japan. However, it somewhat reduces attitudes towards Russia, and exposure to foreigners raises attitudes towards the US and Japan and is not significant for Russia. In addition, those respondents who are more satisfied with their home country’s political society are more likely to have a favourable attitude towards Russia.

Overall, the factors influencing the Chinese public’s attitudes towards the US, Japan, and Russia confirm the discussion of the in-group and out-group relationships described in the previous chapter. These three countries’ relations with China represent three different bilateral relationships and historical patterns. Russia, influenced by various factors over a long period, is seen as an important partner of China in the international community, sharing more similar values and common interests with China, and is seen as belonging to a more identified in-group compared to the US and Japan, which are in a clear out-group position. As a result, the public that identifies more strongly with China tends to have more positive attitudes towards Russia, as indicated by another empirical study by Noel and Decker in 2016 [54]. Furthermore, based on the above findings, it is clear that the factors influencing attitudes towards the US, Japan, and Russia have changed between these ten years. Even the direction of influence of some of the variables has shifted entirely. As this element is not relevant to this paper, we will not expand too much on it here, but it is an issue that deserves further attention in the future. It also confirms that in the eyes of most Chinese public, Russia belongs to the in-group countries, while the USA and Japan belong to the out-group countries.

Next, the model introduces two variables for the weekly frequency of traditional media and Internet media use. According to the regression results, we find that: firstly, for Internet media, the frequency of access to the Internet affects people’s foreign attitudes to some extent in a single year, but not consistently across countries. In 2010, the more frequent the use of Internet media, the more likely it is to have a positive attitude towards the US, and the more likely it is to have a negative attitude towards Japan and Russia; in 2020, the more frequent the use of Internet media, the more likely it is to have a negative attitude towards the US, the more likely it is to have a positive attitude towards Japan, and the effect on attitudes towards Russia is no longer significant. Second, there are differences in the impact of different media channels on Chinese people’s foreign attitudes. According to the findings in Figs 2 and 3, despite the declining use of traditional media during this decade, the traditional media still retains a degree of influence. The impact of traditional media on attitudes towards different countries in the same year is more consistent, with traditional media generating relatively positive attitudes overall in 2010, with more people using traditional media tending to have more positive attitudes towards the US and Japan. In 2020, the impact of traditional media was generally more negative, with people using more traditional media tend to have more negative attitudes towards the US, Japan, and Russia.

## Discussion

Based on the findings mentioned above, it is clear that exposure to different types of media does not have a distinct impact on citizens’ attitudes, except in the case of attitudes towards Japan. In 2010, both the Internet and mass media had a positive impact on citizens’ attitudes towards the US; however, in 2020, the impact of both channels turned negative. As for Japan, where the impact of different media types varied, the results are mixed. In 2010, the frequency of Internet exposure had a negative impact on attitudes towards Japan, while the frequency of mass media exposure had a positive impact. However, in 2020, the opposite was observed, with the frequency of Internet exposure having a positive impact and mass media exposure having a negative impact. Therefore, the frequency of Internet use does not necessarily lead to a deterioration in attitudes towards out-group countries. Moreover, except for 2010, when Internet use negatively affected citizens’ attitudes towards Russia, neither media type had a significant impact. Hence, it can be concluded that the frequency of Internet use does not consistently lead to improved attitudes towards out-group countries.

Compared to 2010, the influence of most media channels also underwent changes in 2020. Regarding attitudes towards the US, both Internet and mass media impact shifted from positive to negative. For attitudes towards Japan, the impact of mass media shifted from positive to negative, while the impact of the Internet changed from negative to positive. As for attitudes towards Russia, the impact of the Internet changed from positive to insignificant, while the impact of mass media remained insignificant in both 2010 and 2020.

Based on the findings and discussions above, the hypotheses have been examined. Given the mixed results between the use of different media types and attitudes, Hypothesis 1, “the more frequent access to the Internet, the more negative attitude towards out-group countries and positive attitude towards in-group countries,” is rejected. On the other hand, Hypothesis 3, “there is no positive relationship between the frequency of Internet access and negative attitude towards out-group countries and positive attitude towards in-group countries,” is accepted. Furthermore, the influence of the Internet on citizens’ attitudes between 2010 and 2020 shows variations that are more aligned with changes in foreign relations. For instance, in 2010, during a period of strained Sino-Japanese relations, an increase in Internet use led to a deterioration of attitudes towards Japan, whereas in 2020, with some short-term improvement in Sino-Japanese relations, increased Internet use improved attitudes towards Japan. Consequently, Hypothesis 2, “the more frequent access to the Internet, the greater the level of consistency in attitudes towards foreign countries,” is rejected. Hypothesis 4, “there is no negative relationship between the frequency of Internet access and attitude change," is accepted.

The examination of the hypothesis demonstrates that the spread of the Internet has not significantly increased nationalist sentiment among the Chinese people. In other words, the increase in the frequency of Internet use has not resulted in a uniformly hostile attitude towards out-group countries and a solely friendly attitude towards in-group countries. Additionally, the impact of increased Internet use on external attitudes is not fixed; rather, non-essential changes in external relations still influence people’s attitudes towards foreign countries. From the perspective of media exposure and citizens’ attitudes towards foreign countries, this paper contributes to the debate on the relationship between the spread of the Internet and the prevalence of popular nationalism. The findings support the viewpoint that the Internet’s influence on Chinese popular nationalism is limited [45–50]. Furthermore, as argued by Duan and Wang, the Internet can be viewed as a pluralistic environment more responsive to external changes, and its impact cannot be generalized [49,50]. This paper also explores the complexity of the Internet’s influence on attitudes. The results demonstrate that Internet exposure does not lead to a worsening of attitudes towards out-group countries, nor does it result in a positive attitude towards in-group countries. Therefore, it is challenging to claim that the Internet serves as the breeding ground for popular nationalism.

Furthermore, understanding the attitudes towards China’s significant opponents and partners, such as the US, Japan, and Russia, holds important implications for gauging public opinion changes. This paper also assessed Chinese citizens’ attitudes towards these countries in both 2010 and 2020. For the US, China’s most regarded opponent, the positive attitude of Chinese citizens decreased from 59.9% in 2010 to 27.6% in 2020, reflecting the deterioration of Sino-US relations since the outbreak of the trade friction in 2018. Conversely, Chinese citizens’ attitude towards Japan, which has been a past adversary and current opponent, increased slightly from 38.3% in 2010 to 40.2% in 2020. This change corresponds to the improvement in Sino-Japanese relations, which was influenced by the alienation of US-Japan relations during the Trump administration. Finally, the deepening of the Sino-Russia relationship, driven by the intensification of China-US strategic competition, contributed to Chinese citizens’ attitudes increasing from 73.6% in 2010 to 84.9% in 2020. Thus, it can be observed that citizens’ attitudes towards foreign countries align with the changes in foreign relationships.

## Conclusion

With the spread of the internet in China, there is increasing concern from foreign observers that the internet will breed popular nationalism and cause growing xenophobia. By utilizing two data from the “Chinese View of the European Union (EU)” project conducted in 2010 and 2020, this paper analysed the influence of the internet on Chinese citizens’ foreign attitudes. The research results conclude that the internet’s spread did not breed the growing popular nationalism. Specifically, the increasing frequency of internet use does not cause the formation of unified negative attitudes towards out-group countries, nor does it produce positive attitudes towards in-group countries. Besides, the increase in internet use frequency did not cause the consolidation of foreign attitudes. The international environment shapes the influence of internet access on the masses’ foreign attitudes to a greater extent. In other words, As the deterioration of attitude towards the US demonstrates, when the bilateral relationship worsened, the attitude towards that country also worsened. As a result, the internet is not the breeding ground for popular nationalism in China, nor does the spread of the internet and the increase in the using frequency of the internet cause xenophobic sentiment.

This paper examined the influence of internet contact on the masses’ foreign attitudes mainly from a macro perspective. That is whether the increase in the frequency of internet use will cause a change in the masses’ attitude towards foreign countries. Future research should be conducted on the micro level by focusing on the content analysis of a specific hot topic on the internet in the period to investigate the nature of information transmitted there and to what extent it is close to nationalism or cosmopolitanism. On the other hand, it is also possible to examine the change in attitude after conducting different kinds of information by using the experimental method to research the influence and mechanism of that influence of information that contains nationalist content. In addition, another limitation of this study is the use of cross-sectional data making any findings of the correlation between variables only tentative, which does not accurately capture the dynamics between 2010 and 2020. Although the process of empirical analysis in this study is sound and based on previous findings, we look forward to more relevant studies coming out in the future to add to the conclusions.

## Supporting information

S1 Table Distribution of variables. (DOCX)

S1 File Data of Chinese View on European Union, 2010. (XLS)

S2 File Data of Chinese View on European Union, 2020. (XLS)
