# Supplementary material for: Did the popularization of the Internet impact Chinese citizens’ attitude towards foreign countries? An empirical study based on two surveys
Source: PLoS One. 2023 Sep 20;18(9):e0291091. doi: 10.1371/journal.pone.0291091 (PMC10511065; doi:10.1371/journal.pone.0291091)
Supplement: S1 Table — (DOCX) [file pone.0291091.s001.docx]

**S1 Table. Distribution of Variables**

|  | **Mean** | | **Standard deviation** | | **Minimum value** | | **Maximum value** | |
| --- | --- | --- | --- | --- | --- | --- | --- | --- |
|  | **2020** | **2010** | **2020** | **2010** | **2020** | **2010** | **2020** | **2010** |
| Gender | 0.539 | 0.504 | 0.499 | 0.500 | **0** | **0** | **1** | **1** |
| Education (in years) | 14.64 | 12.42 | 2.942 | 3.247 | 3 | 0 | 23 | 22 |
| Party membership | 0.112 | 0.235 | 0.315 | 0.424 | 0 | 0 | 1 | 1 |
| Social status scale | 5.378 | 3.866 | 1.592 | 1.638 | 1 | 1 | 10 | 10 |
| National identity | 3.715 | 3.543 | 0.544 | 0.628 | 1 | 1 | 4 | 4 |
| Trust other nations | 2.469 | 2.612 | 0.742 | 0.634 | 1 | 1 | 4 | 4 |
| Political and social satisfaction | 2.856 | 2.395 | 0.534 | 0.6523 | 1 | 1 | 4 | 4 |
| Overseas Experience | 0.206 | 0.163 | 0.404 | 0.369 | 0 | 0 | 1 | 1 |
| Experience of contacting with foreigners | 0.297 | 0.326 | 0.457 | 0.469 | 0 | 0 | 1 | 1 |
| Mass media uses | 1.335 | 3.337 | 1.123 | 1.752 | 0 | 0 | 6.667 | 7 |
| Internet media uses | 5.047 | 3.112 | 1.907 | 2.983 | 0 | 0 | 7 | 7 |
